# An oral lipidic native testosterone formulation that is absorbed independent of food

**DOI:** 10.1530/EJE-21-0606

**Published:** 2021-08-11

**Authors:** John Newell-Price, Hiep Huatan, Jo Quirke, John Porter, Eleni Daniel, Enis Mumdzic, Bernard Voet, Brian Keevil, Martin J Whitaker, Richard J Ross

**Affiliations:** 1 Department of Oncology and Metabolism, University of Sheffield, Sheffield, UK; 2 Diurnal Ltd, Cardiff, UK; 3 Sheffield Teaching Hospital, Sheffield, UK; 4 Department of Clinical Biochemistry, Wythenshawe Hospital, Manchester University NHS Foundation Trust, Manchester, UK

## Abstract

**Context:**

There is no licensed oral native testosterone (NT) because of challenges in the formulation. Licensed oral formulations of the ester, testosterone undecanoate (TU), require a meal for absorption and generate supraphysiological dihydrotestosterone (DHT) levels.

**Objective:**

To develop an oral NT formulation.

**Design and methods:**

A lipid-based formulation of native testosterone filled into soft-gelatin capsules at 40 mg per capsule was designed with 2 years of stability at ambient temperature. Pharmacokinetic comparison studies of this oral lipidic NT formulation to oral TU were conducted in dogs and hypogonadal men.

**Results:**

In dogs, 40 mg NT was well absorbed under fasted conditions whereas 40 mg TU required a high-fat meal: for NT, the mean fed/fasted AUC ratio was 1.63 and for TU 7.05. In hypogonadal men, fed and fasted NT had similar pharmacokinetics: C_max_ mean 26.5 vs 30.4 nmol/L (769 vs 882 ng/dL), AUC_0–10 h_ 87 vs 88.6 h nmol/L. NT (fed state) showed a testosterone AUC increase of 45% between 120 and 200 mg, and NT 200 mg gave a similar mean AUC_0–10 h_ to TU 80 mg: 87 vs 64.8 h nmol/L. Serum TU levels were variable and on a molar basis were ~ten-fold higher than serum testosterone levels after TU 80 mg fed. The DHT: testosterone AUC_0–10 h_ ratio was more physiological for NT than TU being 0.19 vs 0.36. There were no emerging safety concerns with NT.

**Conclusion:**

This novel oral lipidic native testosterone formulation has potential advantages over oral TU of dosing independently of food and a lower risk of supraphysiological DHT levels.

**Significance statement**

There is no licensed oral testosterone because of challenges in formulation, and the oral formulations of the ester, testosterone undecanoate, require a fatty meal for absorption and generate supraphysiological dihydrotestosterone levels. We have overcome the design challenges and formulated an oral native testosterone that can be taken with or without food and provides physiological levels of testosterone and dihydrotestosterone in hypogonadal men. This formulation, DITEST, has the potential advantage of being oral for patients who do not tolerate injections and less risk of adverse events that might theoretically be associated with elevated dihydrotestosterone levels. Future studies will need to define the dosing regimen for replacement in hypogonadal men.

## Introduction

Testosterone was isolated, named, and synthesized in 1935 (1), but to date, no oral native testosterone has been licensed for testosterone replacement therapy. The reason being that oral native testosterone, although absorbed through the intestine, undergoes extensive pre-systemic metabolism along the gastrointestinal tract (2), as well as rapid first-pass metabolism in the liver (3). The oral absorption of testosterone is also dependent on the dosing vehicle, wherein a lipophilic vehicle may increase the proportion of testosterone absorbed via the lymphatic route (4). It is thus difficult to achieve adequate bioavailability of testosterone in order to maintain consistent physiological testosterone levels via the oral route. To address this, different routes of administration for testosterone have been used and native testosterone replacement therapy has been licensed as implants, transdermal, transbuccal, and intranasal therapies (5).

Oral 17α-alkylated androgens such as methyltestosterone and oxymetholone were proved to be effective androgen replacement therapies but were associated with severe liver damage including the development of jaundice, peliosis hepatis, and liver tumours (6). This toxic effect on the liver appears to be specific to oral modified (i.e. non-native) testosterones, particularly methylated testosterone and was not seen with native testosterone in animal models assessing liver toxicity (7). Testosterone undecanoate (TU) is an ester prodrug of testosterone and has a mid-chain length fatty acid at the 17β position and when given orally undergoes absorption in part through the intestinal lymphatic pathway, so circumventing some of the first-pass metabolism through the liver (4). Oral testosterone undecanoate is presented as an oily capsule and has been available in Europe since the 1970s (1); however, TU has to be taken with a meal two or three times daily, has an unpredictable absorption pattern, and generates high dihydrotestosterone (DHT) to testosterone ratio (8, 9, 10). An oral self-emulsifying formulation of TU has recently been approved in the US (Jatenzo^®^, Clarus Therapeutics Inc., USA). The formulation promotes solubilization and intestinal lymphatic absorption of the lipophilic testosterone ester. Deesterification of TU by nonspecific esterases in liver, blood, and tissue results in the production of testosterone. The liberated undecanoic acid moiety is metabolized via beta-oxidation. 5-Alpha reduction of testosterone undecanoate in the gut produces dihydrotestosterone undecanoate (DHTU) and DHT (11). The testosterone undecanoate formulation has to be taken with food, patients have higher than normal DHT levels on treatment and the label is associated with a black box warning regarding an increase in blood pressure (12). These data support the need for new developments in this area.

Various oral formulations of native testosterone have been tested in man although none have been licensed (13, 14, 15, 16, 17, 18, 19, 20). Soon after testosterone’s identification and characterization, oral testosterone administration was disregarded as a viable route of administration and replacement because of poor oral absorption (21). In the 1970s, a micronized form of free testosterone was demonstrated to be absorbed in hypogonadal men but absorption was not reliable enough to progress as therapy (14). Further research, particularly by Amory and coworkers, showed that native testosterone administered as a suspension in oil provided potentially therapeutic levels of testosterone in healthy men (15) and combined with 5α-reductase inhibitors provided physiological testosterone levels both in the fasted and fed state (16). Native testosterone is practically insoluble in water and in fatty oil vehicles (22), and the challenge has been to develop a solution formulation that contains sufficient testosterone concentration to provide reproducible physiological testosterone levels in hypogonadal men. Building upon the previous observations, we have developed a lipidic solution formulation of native testosterone and have tested it in dogs and humans in the fasted and fed state.

## Subjects and methods

### Formulation

Lipidic native testosterone (NT) formulations were developed and assessed *in vitro* for dispersion behaviour in gastric and intestinal media and for physical stability. A single formulation of NT, DITEST, was selected to take forward into preclinical trials (Table 1). The formulation used digestible lipids (oils with carbon chain length > 10 carbons atoms) with the addition of short-medium chain oils and ethanol as a polar co-solvent to assist with solubilization. The formulation was encapsulated in size 00 soft gelatin capsules with 40 mg per capsule inside an aluminium foil blister pack and was stable for 2 years at ambient temperature (25°C).

**Table 1:** Oral lipidic native testosterone formulation (DITEST).

Ingredient	Grade	Quantity, % (w/w)	Quantity per capsule (mg)	Function
Testosterone	Ph. Eur.	5.43	40.0	Active ingredient
Sesame oil	Ph. Eur.	41.39	305.0	Carrier
Propylene glycol monolaurate	Ph. Eur.	31.62	233.0	Surfactant
Benzyl alcohol	Ph. Eur.	16.29	120.0	Solvent
Ethanol	Ph. Eur.	5.27	38.83	Solvent
Gelatin	Ph. Eur.	–	–	Capsule shell

### Pharmacokinetics in dogs

Female beagle dogs (*n* = 4) received a single oral administration on five separate occasions of either 40 mg NT capsules or 40 mg TU (Andriol^®^ Testocaps, MSD, UK) in the fed and fasted state or NT capsules 80 mg fed. Blood samples were taken at 0.5, 1, 2, 3, 4, 6, 10, 12, and 24 h following each dose administration.

### Pharmacokinetics in hypogonadal men

A single-centre, phase 1b study to compare the pharmacokinetics of NT 120 and 200 mg with TU 80 mg (Andriol^®^ Testocaps, MSD, UK) in adult male participants with primary or secondary hypogonadism (EUDRACT: 2015-004255-46). A higher dose of NT to TU was chosen as NT and was expected to have reduced bioavailability compared to TU based on the preclinical dog studies. Key inclusion criteria were male aged 18–80 years; diagnosis of primary testicular failure or secondary hypogonadism due to known pituitary disease or congenital deficit; BMI > 18 kg/m^2^ and <35 kg/m^2^; testosterone level < 8 nmol/L (232 ng/dL) after washout of current testosterone treatment and normal prostate-specific antigen (PSA). Exclusion criteria included history of cancer, myocardial infarction, or unstable cardiovascular disease, and haematocrit levels > 0.5 L/L (50%) at baseline.

The primary objective was to compare the rate and extent of absorption of testosterone from a single dose of NT with a single dose of 80 mg TU in the fed state following the standard FDA high-fat, high-calorie meal defined as an 800–1000 calorie meal where approximately 50% of total caloric content comes from fat (23). The secondary objectives were to assess the impact of food on the rate and extent of absorption of testosterone from NT and the safety and tolerability of two different doses of NT. The exploratory objectives included assessing the levels of DHT in serum. The study was a randomized, active control, single-dose, two-way crossover study in two cohorts. In each cohort, participants were randomized to one of two treatments with treatments separated by a minimum 7-day washout:

**Cohort 1:** in the fed state with a high-fat meal either a single dose of 120 mg (3 × 40 mg) NT followed by a single dose of 80 mg (2 × 40 mg) TU or a single dose of 80 mg (2 × 40 mg) TU followed by a single dose of 120 mg (3 × 40 mg) NT.
**Cohort 2:** a single dose of 200 mg (5 × 40 mg) NT (fed with a high-fat meal) followed by a single dose of 200 mg (5 × 40 mg) NT (fasted) or a single dose of 200 mg (5 × 40 mg) NT (fasted) followed by a single dose of 200 mg (5 × 40 mg) NT (fed with a high-fat meal).

On each dosing day, samples were taken at –0.5, –0.25 (cohort 1 only), 0, 0.5, 1, 1.5, 2, 2.5, 3, 3.5, 4, 4.5, 5, 5.5, 6, 7, 8, and 10 h for pharmacokinetic (PK) assessment. There was a minimum of 3 months separation between treatments in cohorts 1 and 2.

### Assays

Liquid chromatography with tandem mass spectrometry (LC-MS/MS) analysis for serum testosterone and DHT was performed using a Waters Xevo TQ-S ^TM^ mass spectrometer and a Waters Acquity^TM^ LC system with an electrospray source operated in positive ionization mode. For testosterone, the lower limit of quantitation (LLOQ) was 0.1 nmol/L and the assay was linear up to 40 nmol/L. The inter-assay imprecision was 3.9, 3.9, and 3.1% at concentrations of 0.5, 4.7, and 14.0 nmol/L, respectively. The reference range for adult men aged 18–39 years is 9.2–31.8 nmol/L (24). For DHT, the LLOQ was 0.3 nmol/L, and the assay was linear up to 50 nmol/L. The inter-assay imprecision was 11.2, 8.4, and 5.8% at concentrations of 0.3, 0.9, and 8.3 nmol/L, respectively. The reference range for adult men aged < 65 is 0.8–3.5 nmol/L (25). LC-MS/MS analysis for serum testosterone undecanoate was performed using a Waters Xevo TQ-S Micro^TM^ mass spectrometer and a Waters Acquity^TM^ LC system with an electrospray source operated in positive ionization mode. For TU, the LLOQ was 0.002 nmol/L (1.0 ng/L), and the assay was linear up to 4.38 nmol/L (2000 ng/L). The inter-assay imprecision was 11.2, 8.4, and 5.2% at concentrations of 0.02, 0.18, and 1.53 nmol/L (8.0, 80, and 700 ng/L), respectively. All LCMS instruments are calibrated monthly.

### Statistics in hypogonadal men

PK parameters (C_max_ and AUC) were calculated based on actual sampling times with correction for baseline testosterone that is, by subtraction of the mean of individual pre-dose concentrations. In each cohort, the primary PK endpoints were analysed using an ANOVA model corresponding to a two-way crossover design with fixed effects for sequence, treatment, period, and participant nested within the sequence. The comparison between NT 200 mg and TU 80 mg in the fed state was based on the ANOVA model with treatment as the only fixed effect. The analyses were based on the log-transformed concentrations. The 90% CIs for the ratio of the treatment effects were calculated using the mean square error from the ANOVA models. PK parameters (C_max_ and AUC) from the non-compartmental analysis were cross-correlated with body weight.

### Ethics

The study protocol was approved by the North West – Greater Manchester South Research Ethics Committee (Reference number: 16/NW/0242: 193020) and the Medicines and Healthcare Products Regulatory Agency (MHRA), UK. The trial was performed in accordance with the ethical principles that have their origins in the Declaration of Helsinki (October 2013) and in accordance with International Conference for Harmonisation Good Clinical Practice (ICH GCP) with all subjects providing written informed consent.

## Results

### Pharmacokinetics in dogs

Baseline corrected quantifiable testosterone concentrations were reported in all animals (*n* = 4) on all dosing occasions up to at least 4 h after dosing with NT and TU. For NT, systemic exposure of testosterone approximately doubled following an increase in the administered dose from 40 to 80 mg fed, suggesting dose proportionality. TU was poorly absorbed when fasted: geometric mean AUC h ng/mL fasted vs fed 10.7 vs 64.6 whereas NT was absorbed fasted with less difference between geometric mean AUC fasted vs fed 15.4 h ng/mL vs 25.5 h ng/mL (Table 2). The ratio (90% CI) fed: fasted for AUC was 1.63 (1.19–2.07) for NT vs 7.05 (5.79–8.31) for TU.

**Table 2:** Pharmacokinetic parameters for NT and TU in dogs.

Formulation	Testosterone dose (mg)	Fasted or fed	T_max(obs)_ (h)*	C_max(obs)_ (ng/mL)^†^	AUC_0-t_ (h ng/mL)^†^
NT	40	Fasted	0.50 (0.50, 1.00)	7.98 (32.9)	15.4 (16.6)
NT	40	Fed	0.50 (0.50, 0.50)	11.0 (35.3)	25.5 (26.8)
NT	80	Fed	1.00 (1.00, 3.00)	18.7 (38.1)	63.3 (20.6)
TU	40	Fasted	1.50 (1.00, 10.0)	1.78 (45.3)	10.7 (25.4)
TU	40	Fed	2.00 (1.00, 10.0)	18.0 (83.8)	64.6 (20.0)

^*^Values are median (range); ^†^Values are geometric mean (CV%).

### Demographics of hypogonadal men

**Figure 1:**
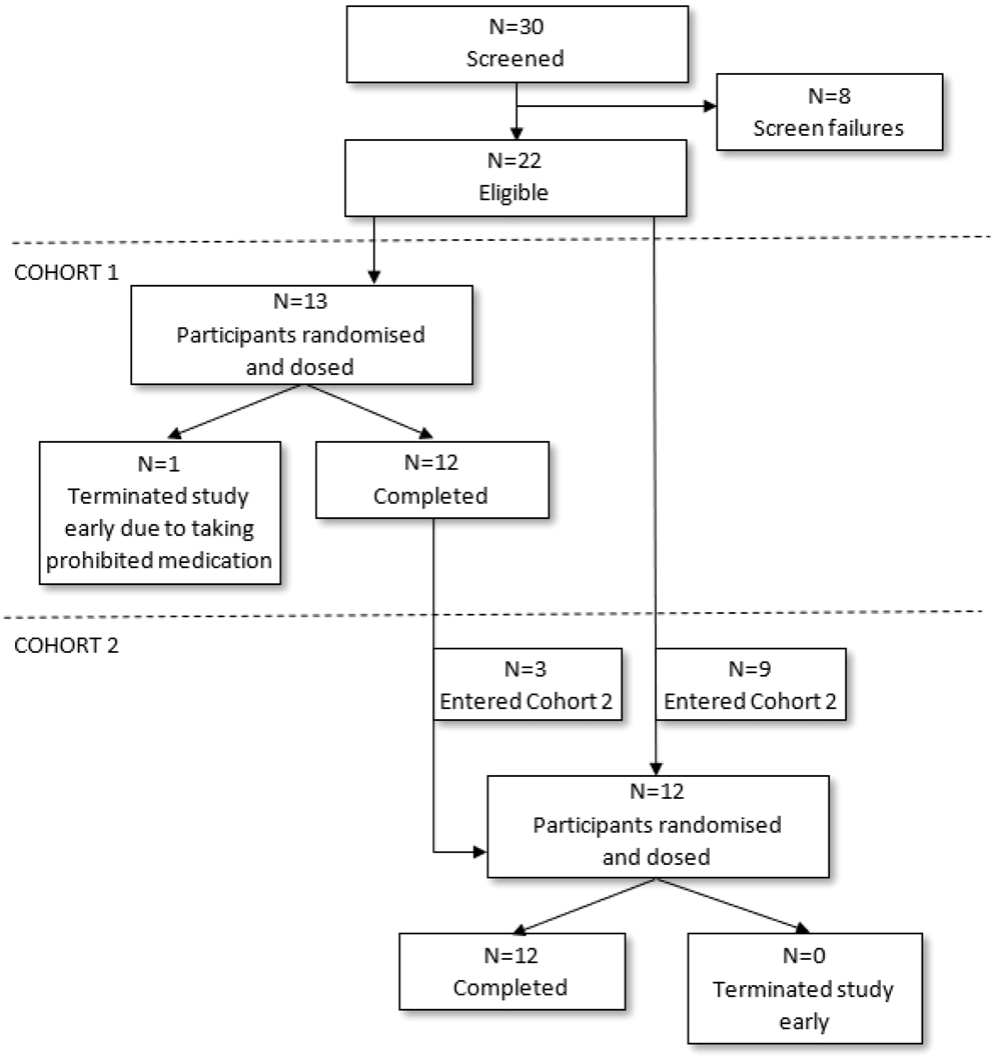
CONSORT diagram, disposition of participants.

A total of 30 participants were screened, with 8 participants failing screening and not taking part in the study (Fig. 1). The reasons for screen failure were testosterone level > 8 nmol/L (232 ng/dL) (*n = *5), haematocrit > 0.5 (*n = *1), BMI > 35 kg/m^2^ (*n = *1) and unable to consume the standard high-fat breakfast (*n = *1). A total of 22 participants were enrolled in the study (in either cohort 1, cohort 2, or both cohorts) and received at least one study intervention. Three participants were enrolled in both cohorts since participants from cohort 1 could be entered into cohort 2 after a washout period of at least 3 months between cohorts. For the purposes of the analysis, these three participants were handled as separate participants in each cohort so a total of 25 individual cases were randomized and treated during the study (Fig. 1). In the overall safety set (*n = *25), participants had a mean (s.d.): age of 53.8 (13.9) years; body weight of 91.7 (13.0) kg; BMI of 29.1 (3.7) kg/m^2^. Most participants were white (92.0%). Mean ± s.d. baseline serum testosterone was 3 ± 2.6 nmol/L (87 ± 75 ng/dL) (Table 3). One participant in cohort 1 was withdrawn early from the study because he started a prohibited medication during the washout between treatment periods. This participant only received the study intervention in period 1 (TU) and was replaced. Twelve participants completed the study in each cohort.

**Table 3:** Baseline demographics.

Cohort	Cohort 1			Cohort 2			Overall (*n = *25)
	Sequence 1 (*n = *6)	Sequence 2 (*n = *7)	All (*n = *13)	Sequence 1 (*n = *6)	Sequence 2 (*n = *6)	All (*n = *12)	
Age, years							
Mean (s.d.)	45.8 (9.2)	57.3 (18.7)	52 (15.7)	58.2 (15.7)	53.3 (7.5)	55.8 (12.0)	53.8 (13.9)
Min-Max	34–60	35–78	34–78	36–72	44–61	36–72	34–78
Height, m							
Mean (s.d.)	1.81 (0.04)	1.76 (0.05)	1.79 (0.05)	1.75 (0.04)	1.77 (0.04)	1.76 (0.04)	1.77 (0.05)
Min-Max	1.75–1.88	1.68–1.81	1.68–1.88	1.72–1.82	1.70–1.82	1.70–1.82	1.68–1.88
Weight, kg							
Mean (s.d.)	93.82 (18.73)	87.19 (10.68)	90.25 (14.66)	96.00 (13.42)	90.72 (9.57)	93.36 (11.45)	91.74 (13.04)
Min-Max	70.00–126.00	77.00–108.60	70.00–126.00	73.00–108.80	81.00–106.44	73.00–108.80	70.00–126.00
BMI, kg/m^2^							
Mean (s.d.)	28.36 (4.66)	28.10 (3.54)	28.22 (3.92)	31.36 (3.84)	28.82 (2.42)	30.09 (3.33)	29.11 (3.70)
Min-Max	21.85–35.65	23.50–33.15	21.85–35.65	24.39–34.74	25.51–32.49	24.39–34.74	21.85–35.65

Cohort 1, Sequence 1: 120 mg NT followed by a single dose of 80 mg TU; Cohort 1, Sequence 2: 80 mg TU followed by 120 mg NT; Cohort 2, Sequence 1: 200 mg NT in the fed state followed by 200 mg NT in the fasted state; Cohort 2, Sequence 2: 200 mg NT in the fasted state followed by 200 mg NT in the fed state.

### Pharmacokinetics in hypogonadal men

**Figure 2:**
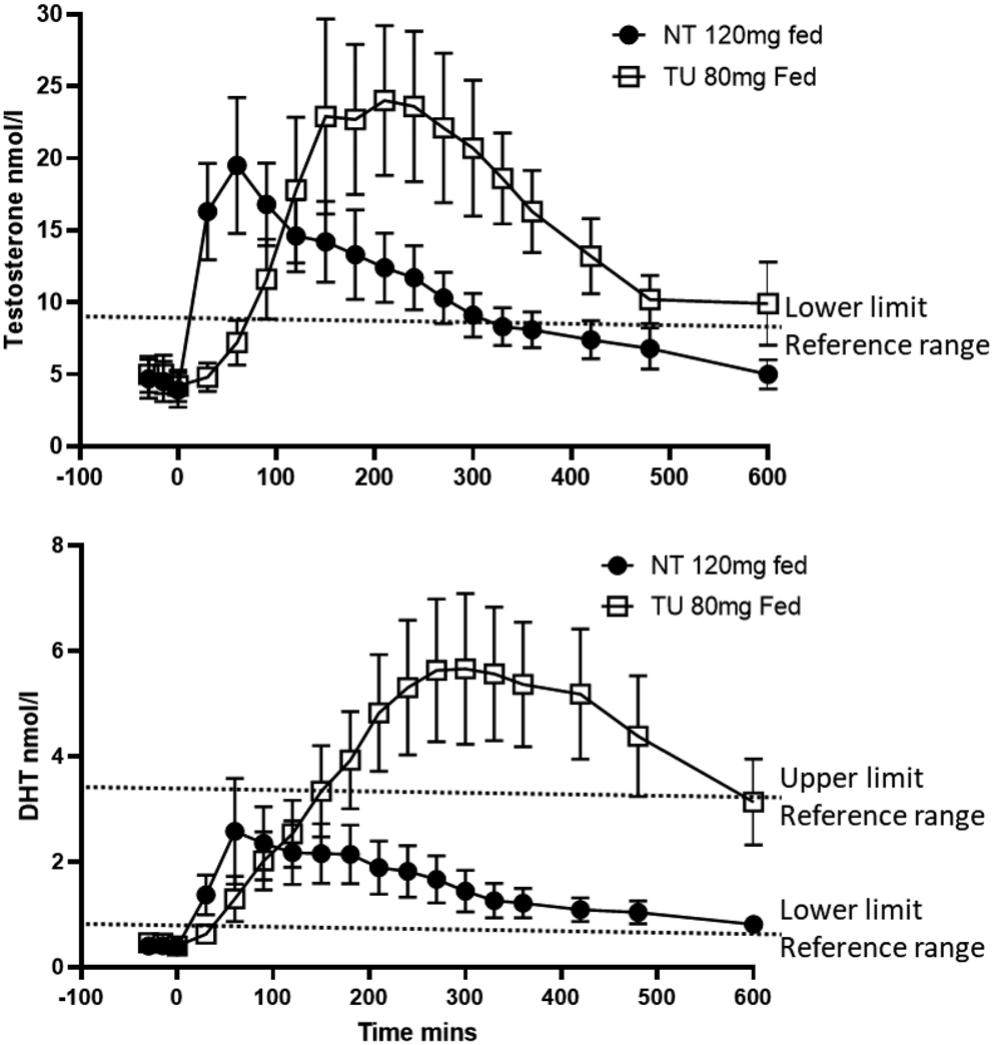
Mean (s.e.m.) serum testosterone and DHT levels following NT 120 mg and TU 80 mg.

**Figure 3:**
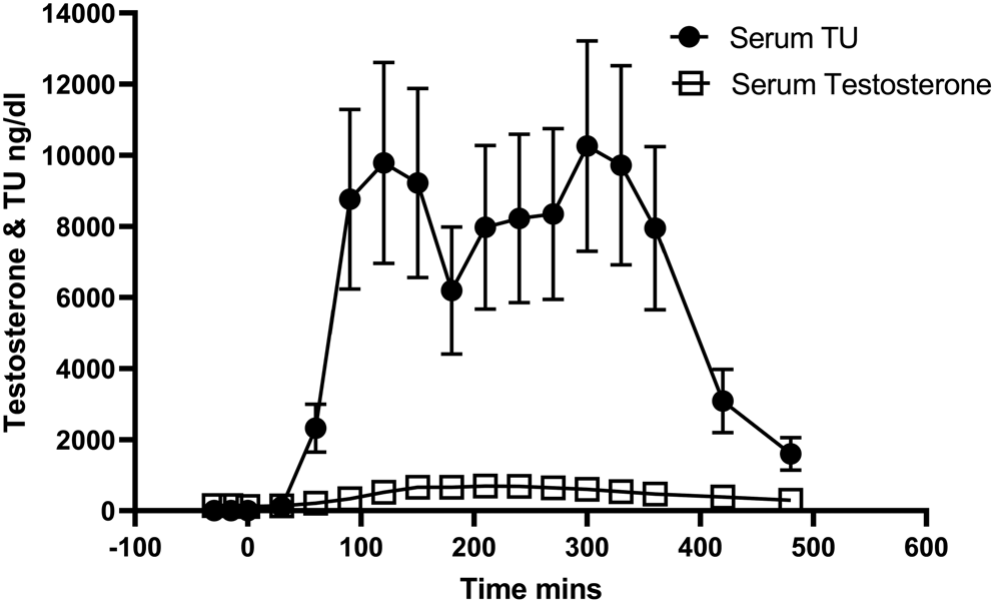
Mean (s.e.m.) serum TU and testosterone levels after TU 80 mg fed.

Cohort 1, comparing 120 mg NT with 80 mg TU taken in the fed state with a high-fat meal showed both formulations generated testosterone levels in the physiological range and 80 mg TU gave higher testosterone levels than 120 mg NT (Fig. 2). NT had an earlier T_max_ than TU: 1.4 vs 4.2 h (Table 4). NT resulted in around 50% lower levels of DHT than TU, and the ratio of DHT: T for AUC_0–10 h_ for NT was 0.19 and for TU 0.36. Serum TU levels after dosing with 80 mg TU were approximately ~ten-fold greater than serum testosterone levels on a molar basis and showed considerable variability between subjects (Fig. 3).

**Table 4:** Pharmacokinetic summary of data comparing native testosterone (NT) with testosterone undecanoate (TU) and NT taken fasted and fed in hypogonadal men (baseline-adjusted pharmacokinetic set). Data are presented as mean (s.d.).

	Cohort 1^†^			Cohort 2^‡^		
	DITEST 120 mg	TU 80 mg	GLSM ratio (90% CI)	Fed	Fasted	GLSM ratio (90% CI)
Testosterone						
C_max_						
ng/dL	554 (481)	911 (670)	55.6 (45–68.7)	769 (421)	882 (458)	85.1 (57.5–126.0)
nmol/L	19.1 (16.6)	31.4 (23.1)		26.5 (14.5)	30.4 (15.8)	
AUC_0–10 h_						
h ng/dL	1726 (1578)	2958 (2480)	51.3 (34.7–75.7)	2523 (1615)	2569 (1496)	94.2 (66.1–134.2)
h nmol/L	59.5 (54.4)	102 (85.5)		87.0 (55.7)	88.6 (51.6)	
T_max_ h	1.4 (1.0)	4.2 (2.1)		1.4 (0.6)	1.2 (0.6)	
TU						
C_max_						
ng/dL		20 900 (8500)				
nmol/L		458 (186)				
AUC_0–10 h_						
h ng/dL		47 200 (18500)				
h nmol/L		1034 (405)				
T_max_ h						
DHT		3.8 (2.3)				
C_max_						
ng/dL	84 (99)	194 (119)		119 (58)	131 (61)	
nmol/L	2.9 (3.4)	6.7 (4.1)		4.1 (2.0)	4.5 (2.1)	
AUC_0–10 h_						
h ng/dL	319 (351)	1053 (850)		467 (270)	484 (299)	
h nmol/L	11.0 (12.1)	36.3 (29.3)		16.1 (9.3)	16.7 (10.3)	
T_max_ h	2.4 (2.1)	5.7 (1.9)		1.7 (0.6)	1.6 (0.7)	

To convert testosterone nmol/L to ng/dL multiply by 29; To convert DHT nmol/L to ng/dL multiply by 29; To convert TU ug/L to ng/dL multiply by 100; To convert TU multiply ug/L by 2.19 to get nmol/L. ^†^Fed NT 120 mg vs TU 80 mg. ^‡^NT 200 mg fed vs fasted.

GLSM, geometric least squared mean.

**Figure 4:**
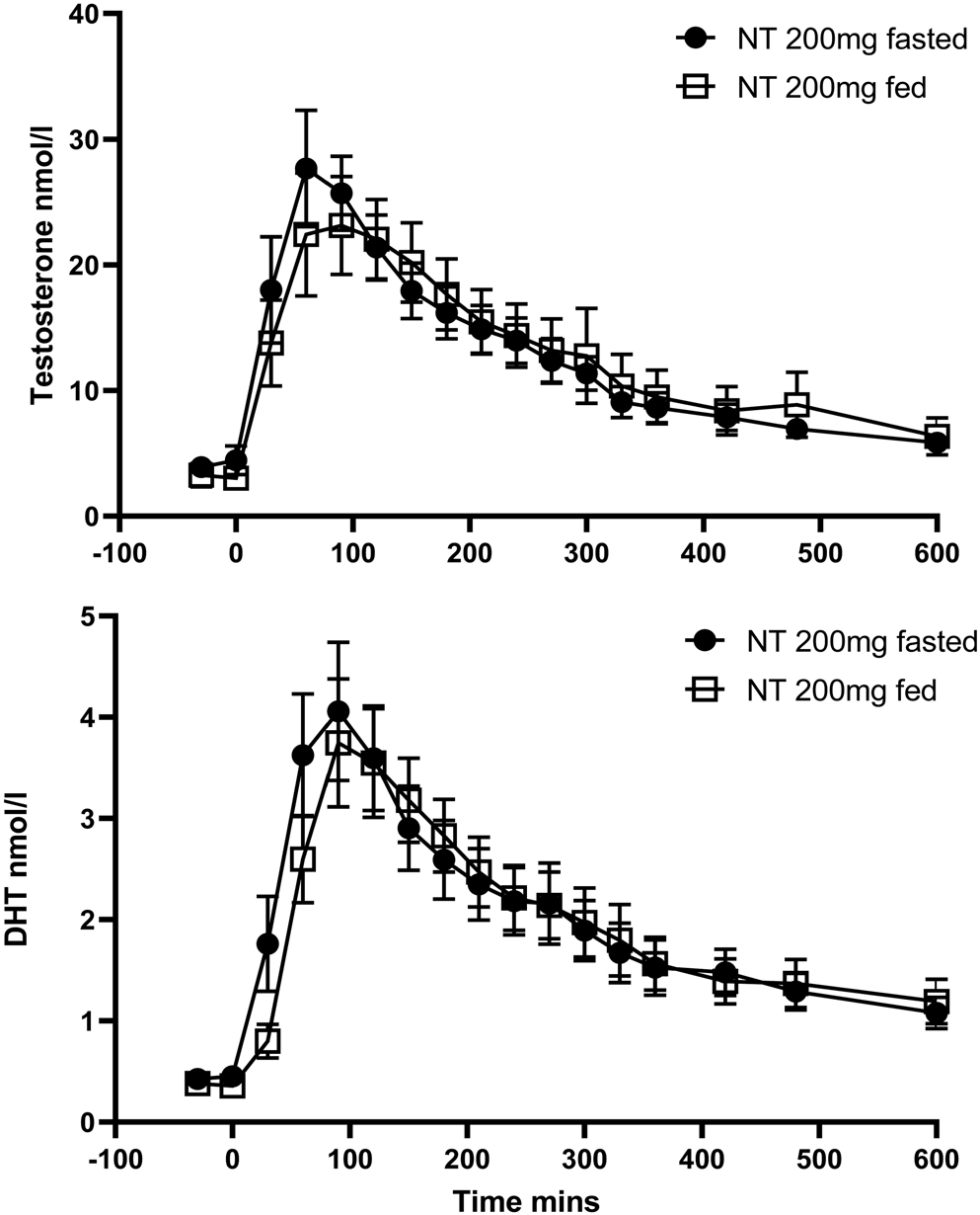
Mean (s.e.m.) serum testosterone and DHT levels following NT 200 mg fasted and fed.

Cohort 2, NT 200 mg given either fed with a high-fat meal or fasted showed similar testosterone levels and pharmacokinetics (Fig. 4). Comparing levels in cohort 2 to cohort 1, NT showed a serum testosterone AUC increase of 45% between 120 and 200 mg. NT 200 mg fasted gave equivalent C_max_ and AUC_0–10 h_ to TU 80 mg fed: 90% CIs 88.0 (58.2–133.1) and 87.5 (54.6–140.2).

Cross correlation of the PK parameters C_max_ and AUC_inf_ for serum testosterone levels after NT using all doses showed weak negative correlation with body weight: *r* of –0.45 and –0.27, respectively.

There was one serious adverse event (urinary retention) during TU dosing. There were no emerging safety concerns, and adverse event frequency and severity were similar between the different treatment arms.

## Discussion

We have developed an oral lipidic formulation of native testosterone in a solution that provides physiological levels of testosterone and DHT when taken with or without food. The preclinical study in dogs showed that the oral lipidic NT formulation showed less variability in absorption between the fasted and fed state compared to TU and that very little TU was absorbed in the fasted state, confirming previous results in the literature (26). The results for the NT formulation were confirmed in hypogonadal men where the NT formulation showed similar pharmacokinetics when taken fasted or fed and the ratio of DHT to testosterone was lower for NT than TU.

It is known that native testosterone is absorbed orally but because of the extensive pre-systemic metabolism in the gastrointestinal tract and rapid first-pass metabolism in the liver, a high dose is required to replace physiological circulating serum testosterone levels (1). This is compounded by the fact that testosterone is practically insoluble in water and fatty acid oils (22), so it has been challenging to generate a solution formulation of testosterone with a testosterone concentration sufficient to replace circulating testosterone levels. We have addressed this by generating a lipidic solution formulation where testosterone is held in solution in the oil phase through the addition of co-solvents: ethanol and benzyl alcohol. The formulation is stable at room temperature for up to 2 years and provides reproducible physiological testosterone levels in hypogonadal men.

TU, an ester prodrug of testosterone, given orally, undergoes absorption through the intestinal lymphatic pathway and thus circumvents first-pass metabolism through the liver. The T_max_ for NT was earlier than TU in both the dog and hypogonadal men reflecting that the NT formulation is likely primarily absorbed via the intestinal transcellular route through the hepatic portal circulation. In the fed state, TU provided higher levels of circulating testosterone per unit dose of testosterone than NT; however, the TU levels of the prodrug in the circulation were ~ten-fold greater than serum testosterone levels on a molar basis and showed great variation. This result is similar to the ~ten-fold greater levels of TU prodrug than total testosterone previously reported for TU (11), suggesting that, although TU is well absorbed, a relatively low fraction is converted to testosterone and most of that conversion probably takes place in the gut at the time of absorption as does the generation of DHT. The FDA-approved TU formulation, Jatenzo^®^, recommends a starting dose of 237 mg (150 mg of unesterified testosterone equivalents based on molecular weight) and a maximum dose of 396 mg (250 mg testosterone equivalents) twice daily (12). These are similar to the dosing levels of the NT formulation used in the phase 1b study, 120 to 200 mg, that provided a physiological testosterone concentration. The recommended starting dose of the European approved TU formulation, Andriol^®^, is 120–160 mg (75–100 mg testosterone) daily and in our study, we found higher testosterone levels after 80 mg Andriol^®^ taken with a high-fat meal than after NT 120 mg. The difference in apparent bioavailability of the different TU formulations may relate to the fat content of the meal in studies or the formulation.

TU requires a fat-containing meal for absorption (8, 9, 10), as illustrated here by very low circulating testosterone levels in the dogs when TU was given fasted. Currently, there is only one marketed US Food and Drug Administration (FDA)-approved TU oral product, Jatenzo^®^, with potentially a second product available shortly, Tlando (Lipocine Inc., US), which has reported conditional approval from the FDA. Jatenzo^®^ was poorly absorbed fasted, TU and DHTU concentrations were 6.2- and 8.8-fold higher, respectively, in the fed state (30% fat meal) compared with fasting (11), and in the phase 3 study was administered twice daily with food (12). In contrast, we have demonstrated that native non-esterified testosterone absorption is not affected by food.

TU formulations that replace physiological testosterone levels generate supraphysiological levels of DHT (13), whereas NT formulations provide more physiological levels of DHT (20). The lipidic NT formulation reported here generated a more physiological ratio of DHT to total testosterone compared to TU. To date, there is no evidence that raised DHT levels are harmful, although theoretically there may be more impact on DHT responsive tissues such as skin and prostate. Reassuringly, however, the increased serum DHT concentrations resulting from therapy with oral testosterone undecanoate were not associated with an increased risk of prostate cancer or prostate enlargement in long-term studies (27). The impact of 17α-alkylated androgens on liver toxicity has not been seen with NT formulations (20), and no change in liver function tests was seen in this single-dose study with the NT formulation. However, longer-term studies with NT are required to examine the impact on liver function. After NT dosing, there was a negative correlation between PK parameters suggesting that the greater the weight the lower the C_max_ and AUC, but the correlation was weak and testosterone replacement is generally titrated according to serum testosterone levels in the individual rather than weight.

This manuscript reports clinical data from a single-dose study in a cohort of hypogonadal men, and future studies will need to generate 24-h pharmacokinetic data at a steady state for a range of dose levels. Consideration will also need to be given to increasing the dose per capsule, measuring SHBG levels, and investigating the potential need for dose titration in clinical practice. Testosterone may induce its own metabolism and so the impact of repeat dosing will need to be examined (14). The levels of testosterone, DHT, and TU were quantified from serum samples, and following the start of the study in hypogonadal males it was recognized that TU can be converted to testosterone in serum *ex vivo* and, therefore, the testosterone levels measured after TU administration may be higher than they would have been if measured in plasma (28).

In conclusion, we have developed a lipidic NT formulation, which when given to hypogonadal men generates similar testosterone and DHT exposure in the fed and fasted state. Compared to published literature on a self-emulsifying formulation of TU at 200 mg (12), the NT formulation at 200 mg provides a similar testosterone C_max_ and no requirement for a meal. This oral lipidic native testosterone formulation has anticipated advantages over current oral therapy of dosing with or without food and a lower risk of supraphysiological DHT levels.

## Declaration of interest

J N P received research funds from Diurnal Ltd; R J R and M J W are Directors; J P and J Q are employees and H H and B V are consultants of Diurnal Ltd.

## Funding

This study was sponsored and funded by Diurnal Ltd. UK.

## Author contribution statement

The first and last authors vouch for the accuracy, completeness of the data and analyses. All authors critically reviewed the manuscript. J N P, H H, J Q, J P, B V, M J W, R J R participated in the design and analysis of the trial. J N P, E D, E M were responsible for recruitment and delivery of the trial. BK was responsible for hormone analysis.
